# Development of a conceptual framework for linking mHealth applications to eRecord systems in Botswana

**DOI:** 10.1186/s12913-021-07134-4

**Published:** 2021-10-15

**Authors:** Kagiso Ndlovu, Maurice Mars, Richard E. Scott

**Affiliations:** 1 Department of Telehealth, School of Nursing & Public Health, College of Health Sciences, University of KwaZulu-Natal, Durban, South Africa; 2 Department of Computer Science, University of Botswana, Gaborone, Botswana; 3 College of Nursing and Health Sciences, Flinders University, Adelaide, South Australia Australia; 4 Department of Community Health Sciences, Cumming School of Medicine, University of Calgary, Calgary, Alberta Canada

**Keywords:** mHealth, eRecord systems, Interoperability framework, Interoperability architecture, OpenHIE, OpenHIM, Botswana, Developing countries

## Abstract

**Background:**

The proliferation of mHealth solutions and eRecord systems is inevitable in developing countries, and ensuring their bi-directional interoperability is essential. Interoperability has been described as the ability for two or more systems or components to exchange information and use the information that has been exchanged. Given the importance of linking mHealth solutions to eRecord systems in the developing world, a suitable interoperability framework is required to provide an agreed approach to interoperability and specify common elements. Although eHealth interoperability frameworks exist in the literature, none meet all the requirements for linking mHealth solutions to eRecord systems in developing countries. The aim of this paper was to describe the design and development of a conceptual framework for linking mHealth solutions to eRecord systems in Botswana, as an exemplar.

**Methods:**

An iterative and reflective process was adopted, supported by existing literature and research including consultations with eHealth experts, and guidance from existing frameworks. These collectively identified key elements, concepts, and standards relevant and essential for framework design and development.

**Results:**

The mHealth-eRecord Interoperability Framework (mHeRIF) was developed which highlights the need for: governance and regulation of mHealth and eRecord systems, a national health information exchange, and which interoperability levels to achieve. Each of these are supported by integral themes and concepts. It also addresses the need for regular review, accreditation, and alignment of framework concepts and themes with a National eHealth Strategy Interoperability Development Process. To demonstrate the framework’s applicability, a proposed architecture for the Kgonafalo mobile telemedicine programme is presented.

**Conclusion:**

Interoperable mHealth solutions and eRecords systems have the potential to strengthen health systems. This paper reports the design and development of an evidence-based mHeRIF to align with, build upon, and expand National eHealth Strategies by guiding the linking of mHealth solutions to eRecord systems in Botswana and other developing countries facing similar circumstances.

## Introduction

eHealth, the “use of information and communication technologies (ICT) for health” [1], is an internationally accepted and promoted need. mHealth, “use of mobile technologies for public health”, [2] has grown to include broad medical and health use and is a component of eHealth that is growing rapidly in both the developed and developing worlds [3–5]. Another component is eRecord systems, which includes Electronic Medical Records (EMR), Electronic Health Records (EHR), and Personal Health Records (PHR). To function efficiently these eHealth components need to interact as seamlessly as possible.

To advance such seamless interaction, interoperability (“the ability for two or more systems or components to exchange information and use the information that has been exchanged [6]”) of mHealth and eRecord systems is urgently required. This is especially so in the developing world, where silos of data have arisen due to ad hoc, often donor driven, initiatives and uncoordinated development [7]. The failure of many implementations could, in part, be due to their lack of interoperability with other eHealth components [8]. Such interoperability can be achieved at various ‘levels’ (technical, syntactic, semantic, organisational and legal) [9–12]. The benefits of interoperable eHealth systems are availability of shared up-to-date information, improved quality of care, and cost savings, while the barriers include cost, security and privacy issues, information overload, and liability issues [12]. mHealth is seen as an important means of offering healthcare services in rural and remote areas and gaining surveillance insight from those same areas. However, this requires bi-directional communication for the information to get into and out of an eRecord system for storage and further use. This requirement is facilitated through an interoperability framework offering an agreed approach to linking mHealth solutions to eRecord systems. Such a framework would allow benefits to be realised, such as improved patient management, quality of care, and decision making, and reduced healthcare costs [8]. The interoperability framework in this paper will facilitate the development of an enabling setting for mHealth interventions and their exchange of information with existing eRecord systems. This is in line with the national vision for establishing a single EHR for Botswana, allowing treatment of patients at any location supported by mHealth solutions [13].

Like many developing countries, Botswana has identified eHealth as a means of improving healthcare provision and delivery [13]. According to Seitio-Kgwokgwe et al., the Botswana national health information system (HIS) has always operated within a very weak policy and regulatory framework characterised by inadequate health information legislation, national policy, and strategic planning [14]. Almost all government clinics and hospitals in Botswana are now connected on the government data network (GDN) at about 2 MBps [15]. Prior to 2013, the Ministry of Health and Wellness (MOHW) had 37 eRecord systems which were later reduced to a manageable nine with particular focus now being on the Integrated Patient Management System (IPMS), District Health Information System 2 (DHIS2), Central Stores Drug Management, and the Patient Information Management System (PIMS) [16].

The recently launched Botswana National eHealth Strategy recognises the need for eHealth interoperability, and states, “Interoperability will be supported by establishing an interoperability architecture/platform that simplifies the complexity of interfaces that will be built between different information systems by creating a mediation layer (Health Information Mediator)” [13]. It also identifies the need for “Establishing a standards and interoperability framework” as well as the need to “Establish a home-grown EHR for Botswana” [13]. However, given the rapid anticipated growth in use of mHealth solutions globally, a notable omission of the strategy is its failure to address mHealth and an interoperability approach or framework for linking mHealth and eRecord systems.

Many developing countries offer public healthcare services across a decentralised network of health facilities. In Botswana these include 3 national referral hospitals, 15 district hospitals, 17 primary hospitals, 357 clinics, 346 health posts and 1117 mobile clinics [13], which account for 98% of healthcare facilities in the country [13]. The health information system landscape in Botswana is characterised by a lack of interoperability within and between the public and private sector eHealth systems, duplication of effort across eRecord systems, manual data sharing, and reporting without standardised procedures, thus posing challenges to confidentiality and loss of patient information [13]. Additionally, medical practitioners have little experience of using eHealth for healthcare provision. Although not sustained, a number of mHealth initiatives have been implemented in Botswana to support priority health programmes through a coalition of public and private partners [17–24]. While these mHealth implementations existed, they were not linked to any eRecord systems. This shortcoming was noted and may have contributed to their demise. The current implementation context for mHealth applications and eRecord systems is barren. In the absence of any formal mHealth services or interoperability in Botswana, this conceptual framework will ensure development of the necessary infrastructural and governance setting to facilitate both implementation and interoperability of future mHealth applications, and allow common data reporting of key health indicators to the MOHW.

A recent review of eHealth interoperability frameworks found none to be entirely suitable nor adequate on their own to address linking of mHealth applications to eRecord systems in the context of the developing world and, more specifically, Botswana [25]. Identified limitations of the frameworks included assumptions of: 1) an adequate pre-existing ICT infrastructure (hardware and software), 2) a health sector architecture utilising established interoperability standards, 3) robust governance structures, 4) a healthcare sector with established eHealth services and human resource capacity to support eHealth systems, and 5) acceptance of ICT solutions by eHealth users including patients. This paper is therefore based on the principle that these ‘assumptions’ will be addressed through the proposed conceptual framework.

Another study, based on local eHealth experts’ opinion and a review of the National eHealth Strategy, described Botswana’s eHealth interoperability landscape and provided guidance on linking mHealth applications to existing eRecord systems [26]. Desirable interoperability features were identified for linking mHealth and eRecord systems, such as interoperability standards, application programming interfaces (APIs), data formats and security considerations. It was also found that the only mHealth implementation recognised by the MOHW, the Kgonafalo mobile telemedicine programme, was not linked to any eRecord system. Kgonafalo was a store and forward mobile phone-based telemedicine programme supporting dermatology, cervical cancer, oral health, and radiology [17]. Interviewees identified four major themes requiring attention: 1) eHealth legislation and governance; 2) eHealth software and infrastructure; 3) data standards, security, and Unique Patient Identifier; and 4) capacity building [26].

Furthermore, the National eHealth Strategy review specifically identifies Free and Open Source Software (FOSS) as a desirable resource that will be pursued. In particular, the Strategy identified the open health information exchange (OpenHIE) framework and its reference tool, the Open Health Information Mediator (OpenHIM), as the preferred approach to support eHealth interoperability [25, 26]. OpenHIE offers an adaptable framework utilising standard-compliant architectural components [27]. The Strategy review further identified the adoption of global goods [28] as an appropriate approach. According to Buchholz et al., a global public good (or global good) is a public good available on a more-or-less worldwide basis [29]. In the digital context, this would refer to universally available software (e.g. FOSS), services and content.

Given the importance of linking mHealth solutions to eRecord systems in Botswana and the developing world, a suitable interoperability framework relevant to their needs is required. Such a framework would provide for an agreed approach to interoperability for organisations wishing to work together, and specify common elements [30, 31]. This aligns with the 2005 WHO World Health Assembly (WHA) WHA58.28 declaration calling for member states to: “consider having long-term strategic plans for developing and implementing eHealth services; acquire health ICT infrastructure appropriate to promote equitable, affordable, and universal access; *and recognise that the lack of a seamless exchange of data within and between health information systems hinders care and leads to fragmentation of systems *[32]. Subsequently, the 2013 WHA66.24 resolution [33] and the 2016 WHO report by the Secretariat [34] advocates for Member States to “consider developing policies and legislative mechanisms linked to an overall national eHealth strategy; *and increase mHealth capacity as it has potential to accelerate progress towards achieving universal health coverage (UHC)*, including ensuring access to quality essential health services”, respectively. Furthermore, the new WHO global strategy on digital health (2020–2025), promotes “syntactic and semantic interoperability according to established norms and standards to enable sharing of information in a connected world” [35].

Building upon prior research findings, the aim of this paper is to describe the design and development of a conceptual framework for linking mHealth solutions to eRecord systems. The resultant mHealth-eRecord Interoperability Framework (mHeRIF) is relevant to both Botswana and the developing world.

## Methods

Although eHealth interoperability frameworks exist in the literature, very little guidance is provided on their design and development, particularly towards linking mHealth solutions to eRecord systems. Similarly, whilst many conceptual frameworks appear in the health-related literature, there is again very little guidance for their design and development. The process adopted in this paper is aligned with published principles and experience [36, 37]. Here, a conceptual framework is considered a visual construct that provides an overall representation and understanding of a network of linked concepts, and their interrelationships, in a coherent structure that identifies key specific elements or activities needed to be developed or implemented to achieve the desired outcome from application of the framework.

The Donabedian Model, a conceptual model that provides a framework for examining health services and identifies structures, processes, and outcomes, was adopted and adapted to establish a structured approach with inputs, processes, and outputs [38]. Inputs were those conditions that existed prior to activities undertaken for this paper’s research, processes were the interpretive and iterative activities undertaken during this paper’s research, and outputs were the products reported as a result of the activities undertaken during this paper’s research.

Input: The conceptual framework for this paper is evidence-based, and developed through literature findings, including consultations with eHealth experts, and existing frameworks. These, collectively, identified key elements, concepts, and standards that are relevant and essential for inclusion [25, 26]. Specific guidance was derived from established frameworks, such as: 1. the OpenHIE framework (a mission-driven Community of Practice including countries, organisations, individuals and donors working to promote sharing of health data across many different software products) [27]; 2. Botswana’s National eHealth Strategy (recommendations for use of open-source frameworks and tools that will be compatible and align with the strategy) [13]; and 3. the Refined eHealth European Interoperability Framework (ReEIF) which splits two of the original interoperability levels (Organisational and Technical) into two sub-levels each (Organisational: Policy and Care Process; Technical: Applications and IT Infrastructure), yielding six levels) [30].

Process: An iterative and reflective process guided framework design and development. Based upon available data sources, key elements were identified, given labels, and grouped. These groups were then mapped and related to one another according to their proximal relationships. A preliminary conceptual framework was devised and its content, elements, groups, relationships, and presentation debated on several occasions by the authors to synthesise and resynthesise the framework until consensus was reached. Over time and several iterations, the framework was progressively transformed.

**Fig. 1:**
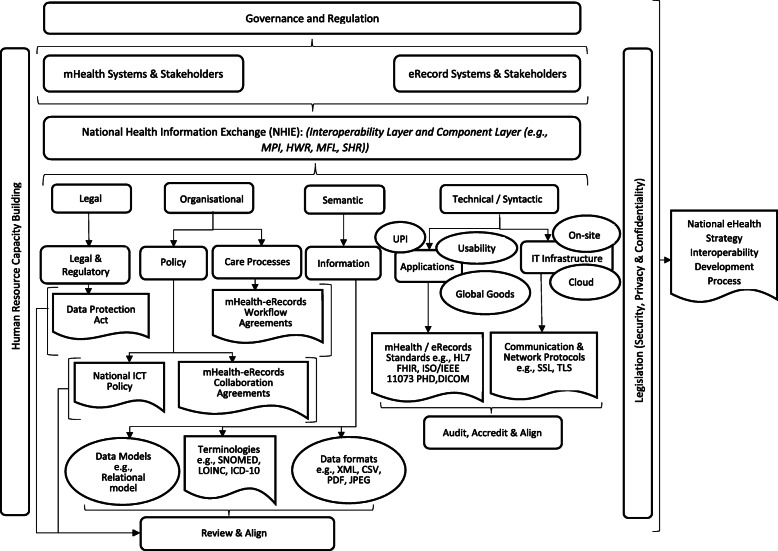
Proposed mHealth to eRecord systems Interoperability Framework (mHeRIF) for Botswana. *Abbreviations / acronyms: CSV* Comma Separated Values; *DICOM* Digital Imaging and Communications in Medicine; *DPA* Data Protection Act; *HL7 FHIR* Health Level 7 Fast Healthcare Interoperability Resources; *HWR* Health Worker Registry; *ICD-10* International Classification of Disease – 10; *ISO/IEEE 11073 (PHD)* International Standards Organisation/Institute of Electrical and Electronics Engineers 11073 (Personal Health Data); *JPEG* Joint Photographic Experts Group; *LOINC* Logical Observation Identifiers Names and Codes; *MFL* Master Facility List; *MPI* Master Patient Index; *PDF* Portable Document Format; *SHR* Shared Health Record; *SNOMED-CT* Systematized Nomenclature of Medicine - Clinical Terminologies; *SSL* Secure Socket Layer; *TLS* Transport Layer Security; *UPI* Unique Patient Identifier; *XML*: Extensible Markup Language

Output: An mHealth-eRecord Interoperability Framework (mHeRIF) was developed (Fig. 1). The framework was then examined by the authors to determine if: key elements from data sources were accommodated, key interrelationships were identified, and the framework provided an adequate visual description, understanding and guidance for linking of mHealth solutions to eRecord systems. Final revisions were made by moving, deleting, or adding elements, adjusting interrelationships, and simplifying visual presentation. As an exemplar, the conceptual framework was used to develop an interoperability architecture for the Kgonofalo mobile telemedicine programme. No further revision to the mHeRIF was required to do so.

## Results

The proposed mHeRIF for Botswana and developing countries is presented in Fig. 1.

At the top of Fig. 1, the framework first illustrates the overarching need for mHealth and eRecord systems governance and regulation which in turn impacts mHealth and eRecords systems and stakeholder coordination, collaboration, compliance with national policies and standards defined within the national health information exchange (NHIE). The framework then illustrates that interoperability will ideally be attained across four distinct levels (Legal, Organisational, Semantic, and Technical/Syntactic). According to the ReEIF these would be further refined into the six sub-layers seen in the next level of the diagram (Legal & Regulation, Policy, Care Processes, Information, Applications, and IT Infrastructure).

Themes, concepts, elements and standards identified from prior studies [25, 26] informed specific details about each interoperability sub-layer. For example, the ‘Applications’ sub layer accommodated the aspects ‘Usability’, ‘Unique Patient Identifier (UPI)’, and the ‘Global goods’ concept. Similarly, the ‘IT Infrastructure’ sub-layer accommodated concepts such as ‘Cloud’ and ‘On-site’ server infrastructures. Other concepts aligned to appropriate sub-layers included the Botswana ‘Data Protection Act’ (DPA), ‘mHealth-eRecord Workflow Agreements’, ‘mHealth-eRecord Collaboration Agreements’, ‘Terminologies’ (e.g. SNOMED-CT, LOINC, ICD-10), ‘Data Models’ (e.g. Relational Data Model), and ‘Data formats’ (e.g. XML, JSON, CSV). Standards, under ‘Applications’, included HL7-FHIR, ISO/IEEE 11073 (PHD), DICOM, while standards under ‘IT Infrastructure’ included Secure Socket Layer (SSL) encryption or Transport Layer Security (TLS) standards. All of these will require regular review, accreditation and alignment to the National eHealth Strategy Interoperability Development Process (far right-hand side of the diagram).

The framework also highlights cross-cutting themes. These include the ‘Human Resource Capacity Building’ (left-hand side of the diagram) and all legislation impacting ‘Security, Privacy and Confidentiality’ concerns (right-hand side of the diagram). All of these feed into a comprehensive and informed ‘National eHealth Strategy Interoperability Development Process’ (far right-hand side of the diagram) supporting linking of mHealth solutions to eRecord systems. This would align with the Botswana Interoperability Pillar outlined in the National eHealth Strategy. Although a generic framework, the proposed mHeRIF may require modest adaptation below the ‘legal, organisational, semantic, and technical’ sub-layer that will allow the enterprise architecture to better suit each specific implementation.

**Fig. 2:**
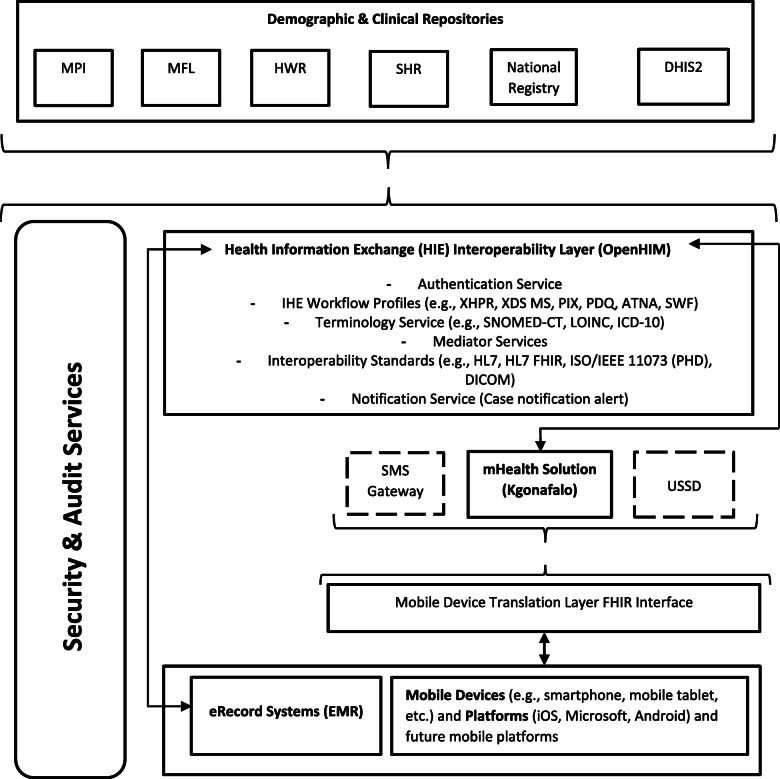
Proposed interoperability architecture design for Kgonafalo mobile telemedicine programme using mHeRIF. *Abbreviations / acronyms: ATNA* Audit Trail and Node Authentication; *DICOM* Digital Imaging and Communications in Medicine; *DHIS2* District Health Information System version 2; *FHIR* Fast Healthcare Interoperability Resources; *HWR* Health Worker Registry; *HL7* Health Level 7; *ICD-10* International Classification of Disease – 10; *IHE* Integrating the Healthcare Enterprise; *ISO/IEEE 11073 (PHD)* International Standards Organisation/Institute of Electrical and Electronics Engineers 11073 (Personal Health Data); *LOINC* Logical Observation Identifiers Names and Codes; *MFL* Master Facility List; *MPI* Master Patient Index; *PDQ* Patient Demographics Query; *PIX* Patient Identifier Cross-referencing; *SHR* Shared Health Record; *SMS* Short Message Service; *SNOMED-CT* Systematized Nomenclature of Medicine - Clinical Terminologies; *SWF* Scheduled Workflow; *USSD* Unstructured Supplementary Service Data; *XDS MS* Cross-Enterprise Document Sharing Medical Summaries; *XPHR* Exchange of Personal Health Record

The current gap in the literature being addressed, and shortfall in Botswana’s (and possibly others’) National eHealth Strategy, is the inability of mHealth solutions to link with eRecord systems. The proposed mHeRIF resolves this shortfall. To demonstrate this, the framework’s functionality was used to propose an OpenHIE compliant architecture for linking the Kgonafalo mobile telemedicine programme to eRecord systems in Botswana (Fig. 2).

The mHealth solution and eRecord system are identified as Point of Service Applications (POSA), linking directly or indirectly with the HIE. The Mobile Device Translation Layer FIHR Interface supports implementation of various mobile devices and platforms (e.g., iOS, Microsoft, Android).

Here, the Kgonafalo mobile solution would be linked to an EMR system through the interoperability layer supporting specific services including the ‘Case Notification Service (CNS)’. The CNS would be responsible for sending bi-directional medical case notifications across mHealth and eRecord systems, for example, when a new case is registered using the mHealth solution and resolved through the eRecord system (e.g., an EMR).

Various repositories (e.g., Master Patient Index (MPI), Master Facility List (MFL), Shared Health Record (SHR)) all work within the OpenHIE framework [27]. The architecture would have the DHIS2 platform as the main repository containing aggregate level content from the various registries. The Integrating the Healthcare Enterprise (IHE) workflow profiles (endorsed by the European Commission [39]) would support the various healthcare scenarios for the Kgonafalo mobile telemedicine programme. The Exchange of Personal Health Record Content (XPHR) integration profile would describe the content and format of summary information extracted from a PHR System for import into an EMR System, and vice versa. The Cross-Enterprise Document Sharing Medical Summaries (XDS MS) profile would describe content and format of discharge summaries and referral notes. Unique Patient Identification across systems would be supported by the Patient Identifier Cross-referencing (PIX) and the Patient Demographics Query (PDQ) Integration Profile. A Scheduled Workflow (SWF) would integrate ordering, scheduling, imaging acquisition, storage and viewing for examinations. The Audit Trail and Node Authentication (ATNA) Integration Profile would support basic security through functional access controls, defined security audit logging and secure network communications.

Specific interoperability standards supporting the proposed IHE profiles would be embedded within the interoperability layer (OpenHIM). The ‘Mediator’ service of the HIE would handle queries and responses between different database systems and resolve complex orchestration of communications between multiple mHealth solutions and eRecord systems. As noted in the methods, the mHeRIF may require modest adaptation for specific services. In Fig. 2, examples are shown within broken line boxes where alternate considerations have been incorporated within this proposed architectural solution for the Kgonafalo mobile telemedicine programme to illustrate this. These were use of an unstructured supplementary service data (USSD) option and the short message service (SMS). Lastly, security and audit services are essential (left-hand side of the diagram) and encompass all of the architecture components. This example demonstrates flexibility in the application of the mHeRIF.

## Discussion

This paper describes the evidence-based design and development of a conceptual interoperability framework (mHeRIF) for linking mHealth solutions to eRecord systems within Botswana and other developing countries in similar circumstances. The proposed mHeRIF emphasises the need for; Governance and regulation of mHealth and eRecord systems, a national HIE supporting all four interoperability levels (Legal and Policy considerations, Shared Care Processes, use of Global goods applications), and an IT infrastructure supporting both cloud and on-site servers (Fig. 1). Furthermore, the mHeRIF highlights the importance of: human resource capacity building; security, privacy, and confidentiality measures aligned to the DPA (or equivalent legislation); as well as standardised data exchange through workflow profiles, terminologies, interoperability standards and common data models. This paper demonstrated use of the mHeRIF to design an architecture for the Kgonafalo mobile telemedicine programme in Botswana.

Growth in the application of mHealth and eRecord systems can be reasonably anticipated, particularly in developing countries [40–42]. The importance of linking these systems is also growing, and has been discussed by others. For example, Hohemberger et al. demonstrated the importance of real-time patient monitoring through wearable devices and mobile applications feeding into hospital or clinic EHRs to support timely decision making [43].

The proposed mHeRIF has the potential to contribute significantly to health system strengthening and a reduction in health expenditure [30, 44]. However, strong political and leadership buy-in have been previously identified as prerequisites for the adoption of interoperable eHealth systems [45–48]. Similarly, political and leadership buy-in are of importance if cloud solutions, not at this point pursued by the MOHW in Botswana, are to support specific user needs of facilities not able to install and maintain sophisticated on-site server hardware [49]. As a consequence, the mHeRIF highlights the overarching need for governance structures to ensure coordination of interoperability activities for various implementation scenarios. Governance and legislation will further ensure adoption and compliance to the mHeRIF by all stakeholders in-line with the Botswana National eHealth Strategy.

The benefits of HIE-based systems in different healthcare settings are well documented [50–52]. In this framework the interoperability layer is supported by the OpenHIE framework which allows flexibility to adapt to country interoperability requirements. This layer allows mHealth solutions to interact with each other and with existing eRecord systems, enabling data sharing across multiple systems. The OpenHIE framework has been previously implemented to support successful interoperability efforts in projects such as the MomConnect mHealth initiative in South Africa [53], and the development of a national concept dictionary for EHR implementation in Kenya [54]. In Asia, most countries are either planning to establish national OpenHIE architectures, or have already partially implemented OpenHIE for a few use cases [55]. HIE workflow profiles within the interoperability layer define shared clinical workflows supporting standardised health information exchange across mHealth and eRecord systems.

The adoption of global goods designed to be interoperable is suggested within the Botswana eHealth Strategy, and is reflected within the mHeRIF. Of importance to their adoption is their usability, to ensure they effectively serve the needs of intended users [56, 57]. Previous studies documented the importance of usability, real-time feedback features, and decision support capabilities in telemedicine systems [56, 58]. A recent study demonstrated how global goods can quickly (within a week) generate a solution for COVID-19 data management [59]. It adopted human-centred approaches focusing on User Acceptance Testing (UAT) throughout the development process to ensure the system meets the needs of target users.

Interoperability standards for mHealth solutions and eRecord systems including the HL7 FHIR, ISO/IEEE 11073 (PHD), and the Digital Imaging and Communications in Medicine (DICOM) have been previously suggested for Botswana and other developing countries [25, 26, 60, 61]. The ISO/IEEE 11073 standard that ensures interoperability between personal health sensors is well documented, and supported by common platforms, like Android [62].

Associated with mHealth and eRecord systems is the use of a unique patient identifier (UPI) crucial to support interactions with the Master Patient Index (MPI) for uniquely identifying patients. In the absence of a national UPI the mHeRIF uses the national identity number for Botswana, the Omang number, or the passport number of foreigners. Use of biometric identifiers could later be introduced to strengthen the uniqueness of patient identifiers [63].

The proposed mHeRIF emphasises security, privacy and confidentiality across all four interoperability layers. A previous study by Rubio et al. implemented varying security measures across the interoperability levels [58]. They further categorised security according to user, agent, manager device and data transfer security. The mHeRIF suggests the DPA (and other legislation as applicable and necessary) be applied to address the necessary safeguards related to security, privacy and confidentiality of personal data in Botswana and across borders.

Although not unique to the developing world, human resource capacity to design, develop and use interoperable eHealth systems is a challenge in Botswana with a previous study showing lack of understanding of the features and interoperability of the eRecord systems and the mHealth application in facilities [26]. This warrants the inclusion of human resource capacity building within the mHeRIF as mandatory.

The need to constantly review, audit, accredit and align all interoperability efforts with the National eHealth Strategy Interoperability Development Process is crucial since new challenges and scenarios will emerge over time requiring different technology approaches. Regular assessment of interoperability models has been recommended to improve their functionality [64]. Similarly, certification of personal health devices using the ISO/IEEE 11073 (PHD) standards should be by a certificate authority to “ensure a high level of protection for human health and safety, smooth operation of the single market and to achieve the results for which the devices are intended” e.g. in the European Union, a medical device is a device certified by the European directive 93/42/CEE) [62]. Currently there is no certification body or legislation in Botswana addressing certification of eHealth devices.

The proposed conceptual framework (mHeRIF) offers developing countries a means to refine their eHealth strategies to ensure inclusion and interoperability of mHealth solutions and eRecord systems. Given the WHO’s promotion of both mHealth and eRecord systems as essential tools [42, 65], this becomes crucial.

## Conclusion

mHealth and eRecords interventions have demonstrated potential to strengthen health systems but their combined leverage has been hampered by approaches that did not consider their interoperability. An interoperability framework adaptable to different implementation contexts will alleviate such challenges by offering organised workflows and standardised health information exchanges, resulting in enhanced eRecord data quality for better health system monitoring and patient care. This paper presents an evidence-based mHeRIF developed using prior research findings as well as guidance from the ReEIF and OpenHIE frameworks. The mHeRIF will align with, build upon, and expand National eHealth Strategies by guiding the linking of mHealth solutions to eRecord systems in Botswana specifically, but also other developing countries facing similar circumstances.

## Data Availability

The data that support the findings of this study are publicly available from the OpenHIE community at https://ohie.org/, the European Union eHealth Network at https://ec.europa.eu/health/sites/default/files/ehealth/docs/ev_20151123_co03_en.pdf and the IHE Europe at https://www.ihe-europe.net/sites/default/files/2018-08/Flyer_27_profiles.pdf. The dataset reviewed in this study (the Botswana National eHealth Strategy) is available from the Botswana Health Data Collaborative repository, at https://ehealth.ub.bw/bhdc/ehealthstrategy.html.

## Abbreviations

ATNA: Audit Trail and Node Authentication
CSV: Comma Separate Values
DICOM: Digital Imaging and Communications in Medicine
DHIS2: District Health Information System version2
DPA: Data Protection Act
eHealth: Electronic Health
EHR: Electronic Health Record
EMR: Electronic Medical Record
eRecord: Electronic Record
FHIR: Fast Healthcare Interoperability Resources
HL7: Health Level 7
HWR: Health Worker Registry
ICD-10: International Classification of Disease - 10
IHE: Integrating the Healthcare Enterprise
ISO/IEEE 11073 (PHD): International Standards Organisation/Institute of Electrical and Electronics Engineers 11073 (Personal Health Data)
JPEG: Joint Photographic Experts Group
LOINC: Logical Observation Identifiers Names and Codes
MFL: Master Facility List
mHealth: Mobile Health
mHeRIF: Mobile Health-Electronic Record Interoperability Framework
MPI: Master Patient Index
OpenHIE: Open Health Information Exchange
OpenHIM: Open Health Information Mediator
PDF: Portable Document Format
PDQ: Patient Demographics Query
PHR: Patient Health Record
PIX: Patient Identifier Cross-referencing
POSA: Point of Service Applications
SHR: Shared Health Record
SMS: Short Message Service
SNOMED-CT: Systematized Nomenclature of Medicine - Clinical Terminologies
SSL: Secure Socket Layer
SWF: Scheduled Workflow
TLS: Transport Layer Security
UAT: User Acceptance Testing
UPI: Unique Patient Identifier
USSD: Unstructured Supplementary Service Data
XDS MS: Cross-Enterprise Document Sharing Medical Summaries
XML: Extensible Markup Language
XPHR: Exchange of Personal Health Record
